# Supplementary material for: Oncogenic structural aberration landscape in gastric cancer genomes
Source: Nat Commun. 2023 Jun 22;14:3688. doi: 10.1038/s41467-023-39263-1 (PMC10287692; doi:10.1038/s41467-023-39263-1)
Supplement: Supplementary file 1 — Supplementary Information [file 41467_2023_39263_MOESM1_ESM.pdf]

(a)

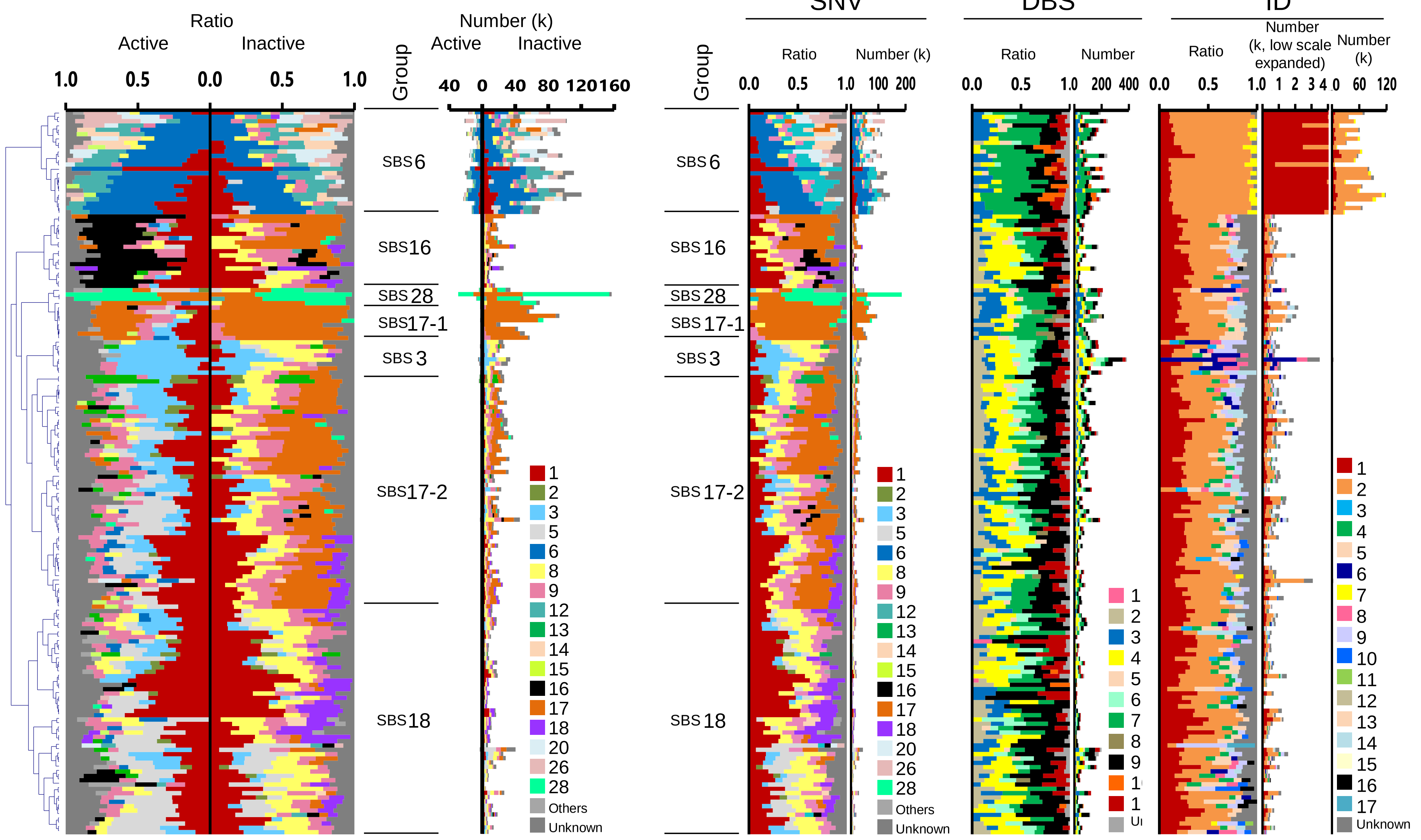

(Figure continued on next page)

(b)

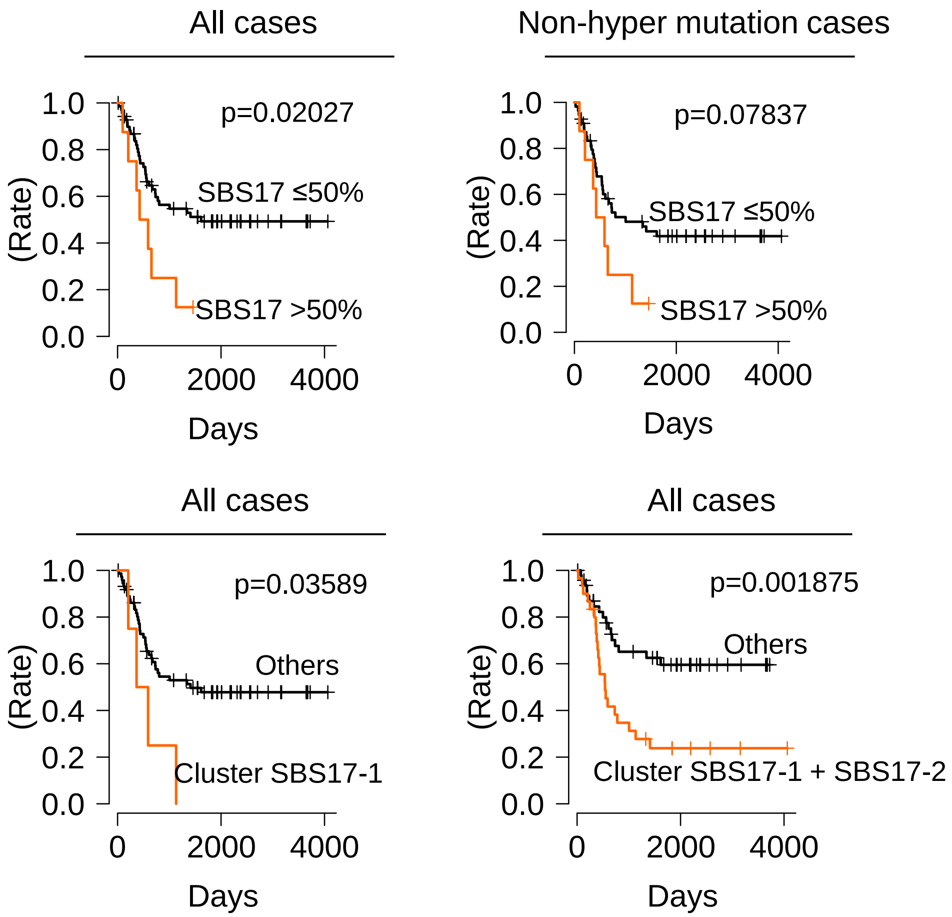

Supplementary Figure 1 Whole-genome mutational signature landscape in gastric cancer (GC). **a.** (Left) Integrated analysis of the chromatin status of stomach mucosa using hierarchical clustering, seven groups exhibiting distinctive contributions of COSMIC single-base substitution signature (SBS) were identified. Two groups appeared for SBS17: one group had a large number of SNVs and a high contribution of SBS17 in both active and inactive areas; the other group did not have such a large number of SNVs and had a high SBS17 contribution only in the inactive area (odds ratio = 7.1, $P < 2.2 \times 10^{-16}$, Fisher's exact test). The contribution of SBS17 was significantly more prevalent in the antrum and smoker ($P = 3.4 \times 10^{-3}$, and $P = 2.6 \times 10^{-3}$ in the inactive area of non-hyper-mutated cases, respectively). SBS28 (unknown) was frequent in both the active and inactive areas in the Epstein-Barr virus (EBV) positive cases ($P = 5.5 \times 10^{-5}$ in the active area, $P = 0.015$ in the inactive area, Fisher's exact test). SBS3 (*BRCA*) was dominant in the active area (odds ratio = 2.4 and $P < 2.2 \times 10^{-16}$, Fisher's exact test).
(Right) SBS3 (*BRCA*) correlated not only with ID6 (R = 0.59 and P = 9.8×10^{-15} in non-hyper-mutated cases, Spearman's rank correlation test), but also ID8 and DBS6 (R = 0.54 and $P = 2.2 \times 10^{-12}$ and R = 0.40 and $P = 7.9 \times 10^{-7}$ in non-hyper-mutated cases, respectively). In the SBS3 group, the DBS number is as high as in the SBS6 (defective MMR) group; however, the count of SNV and ID is less.
b. Survival analysis comparing the SBS17-high group (SBS17 greater than 50%, n = 8) with the SBS-17 low group (< 50%, n = 70). The SBS17-high group had a poor prognosis compared to the SBS17-low group. A similar trend, although not significant, was observed in the non-hyper-mutated cases only (n = 8 and n = 55, respectively, for high and low). In the group of SBS signatures in **a**, the SBS17-1 and the SBS17-2 exhibited poor survivals. P-values are calculated using the log-rank test. **a-b,** Source data are provided as a Source Data file.

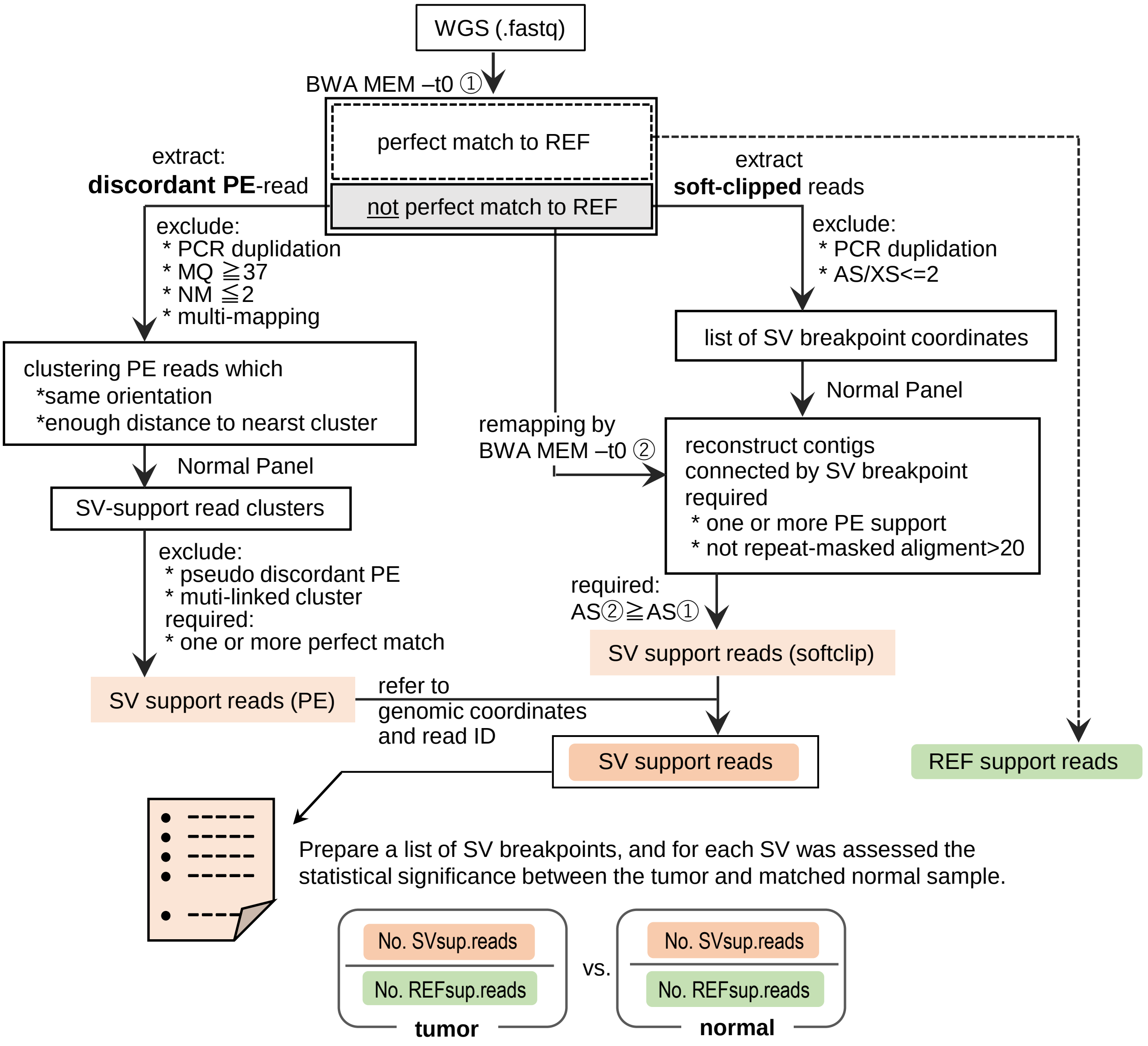

Supplementary Figure 2. Flowchart illustrating the in-house structural variant caller. The flowchart illustrates the in-house structural variant caller. Abbreviations: WGS, whole genome sequence; REF, reference genome (hg19); PE, paired-end; SV, structural variant; MQ, mapping quality score; AS, Alignment score; XS, alternative alignment score; NM, edit distance to the refererence.

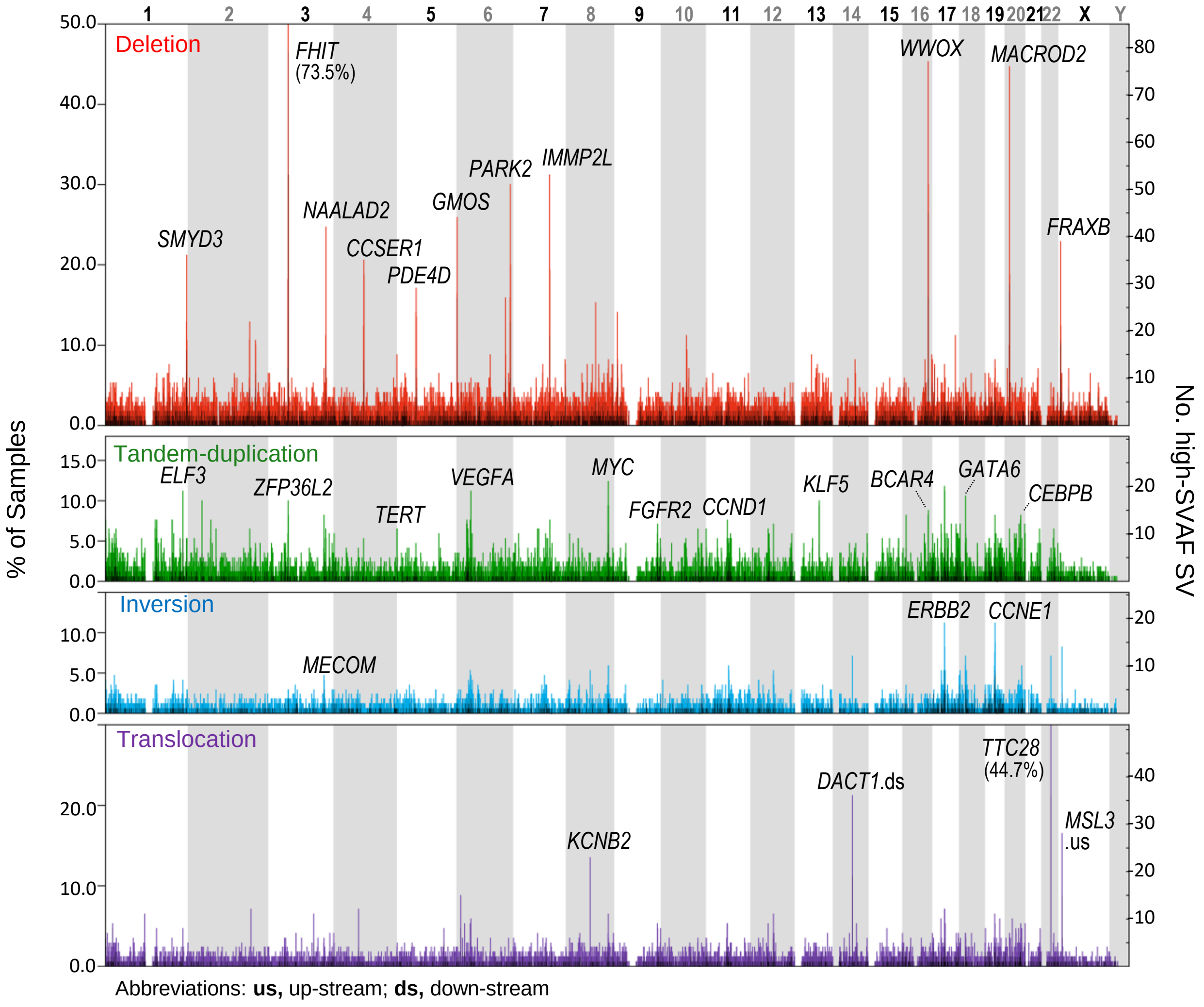

Supplementary Figure 3 Genome-wide hotspot landscape in GC.
The genome-wide frequencies of deletions (red), tandem duplications (green), inversions (cyan), and translocations (purple) were calculated using a sliding window of 500 kb (50-kb overlap). The primary Y-axis shows the percentage of SV-detected cases, and the secondary axis and black histograms indicate the number of SVs with SVAF > 0.4. Source data are provided as a Source Data file.

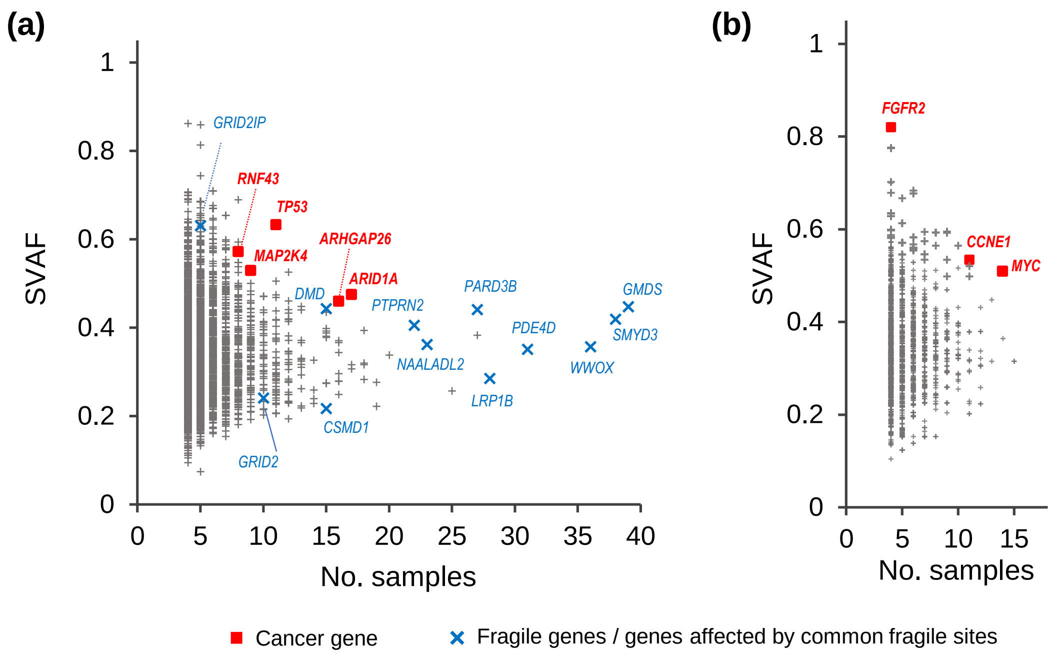

Supplementary Figure 4 Putative GC driver genes show higher SVAF.
a. The median SVAF and number of SV-positive cases plotted for 3,924 genes that contained intragenic SVs (containing at least one exon between its breakpoints), gene fusions, or whole gene deletions. **b**. The median SVAF and number of SV-positive cases plotted for 2,618 genes amplified by tandem duplications. **a-b,** Source data are provided as a Source Data file.

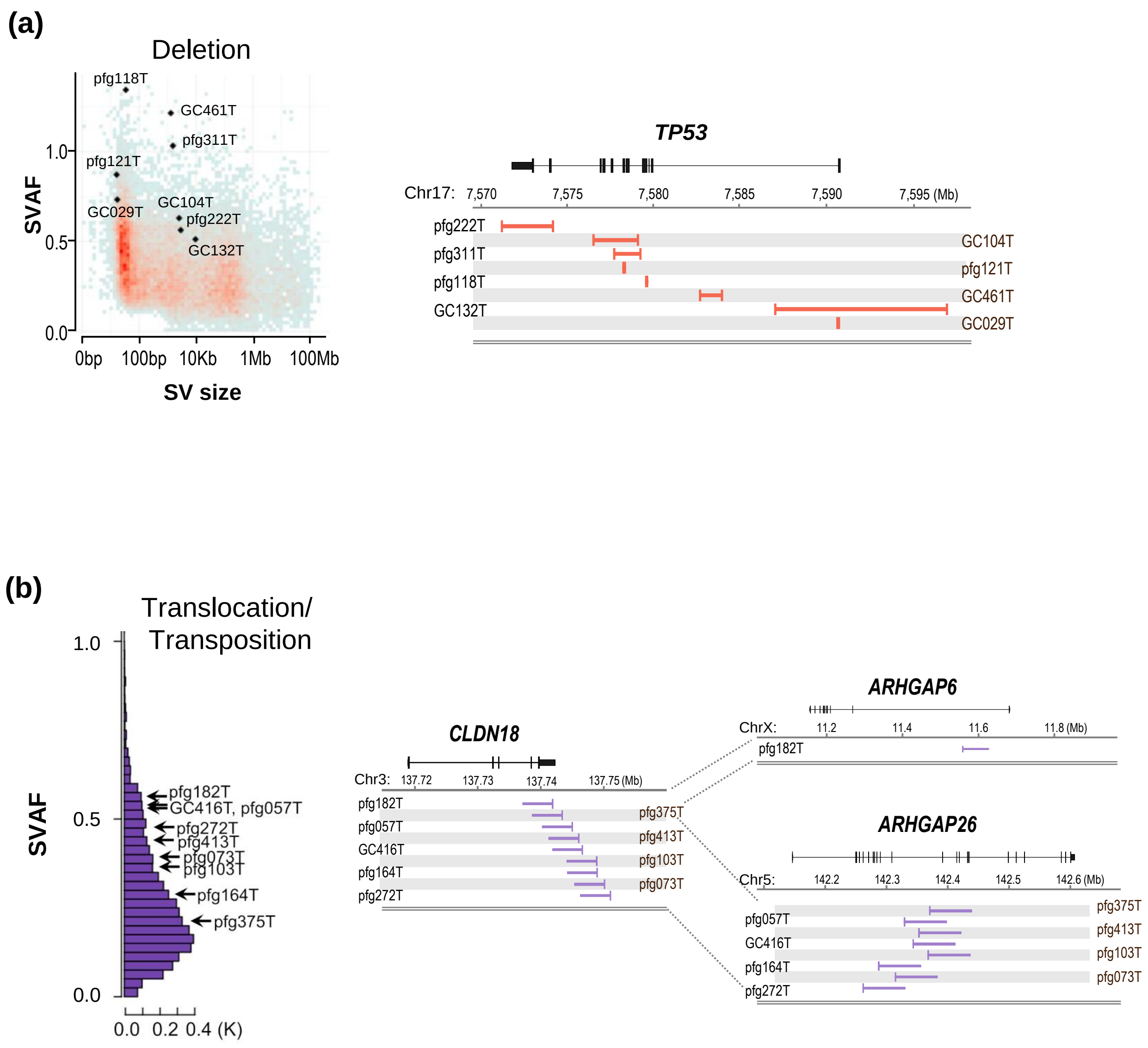

Supplementary Figure 5 Oncogenic gene dysfunction caused by SVs.
The SVs disrupting gene function were marked in the SV size-SVAF plot (or SVAF distribution histogram) and the SV distribution in the representative gene loci are shown.
a. Deletions on *TP53* loci, **b**. Translocation forming *CLDN18-ARHGAP* fusion gene, **c**. Complex SV cluster in the *CCNE1* locus. Tandem duplications and inversions concentrated in the *CCNE1* locus (top panel). Expression of *CCNE1* and nearby genes increased in SV-positive cases (bottom right panel). **a-c,** Source data are provided as a Source Data file.

(c)

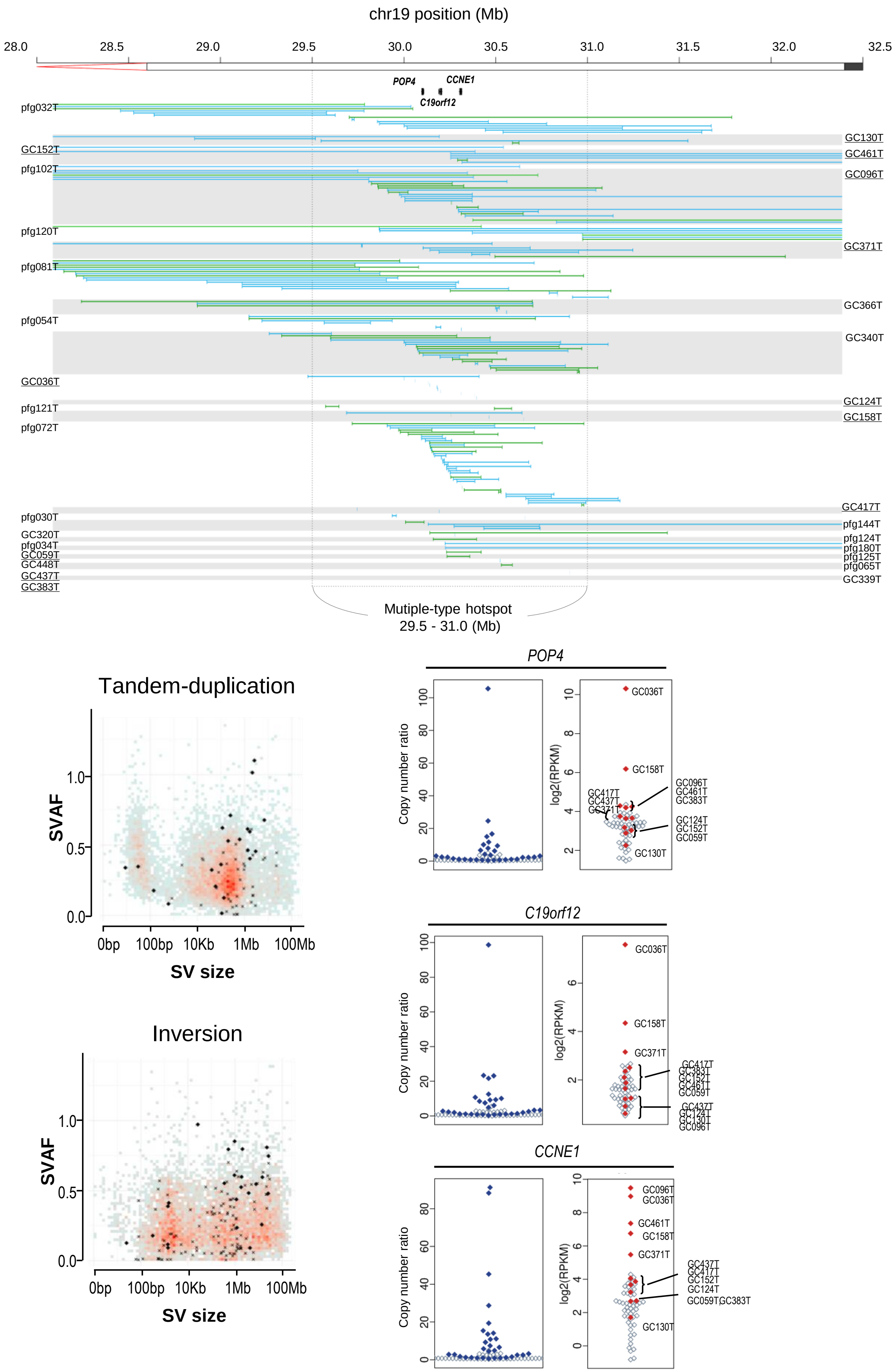

chr19 position (Mb)
28.0
28.5
29.0
29.5
30.0
30.5
31.0
31.5
32.0
32.5
POP4
CCNE1
C19orf12
pfg032T
GC130T
GC152T
GC461T
pfg102T
GC096T
pfg120T
GC371T
pfg081T
GC366T
pfg054T
GC340T
GC036T
GC124T
pfg121T
GC158T
pfg072T
GC417T
pfg030T
pfg144T
GC320T
pfg124T
pfg034T
pfg180T
GC059T
pfg125T
GC448T
pfg065T
GC437T
GC339T
GC383T
Mutiple-type hotspot
29.5 - 31.0 (Mb)
Tandem-duplication
SVAF
SV size
0bp
100bp
10Kb
1Mb
100Mb
Inversion
POP4
C19orf12
CCNE1
Copy number ratio
log2(RPKM)
GC036T
GC158T
GC371T
GC096T
GC461T
GC383T
GC417T
GC437T
GC124T
GC152T
GC059T
GC130T
GC059T,GC383T

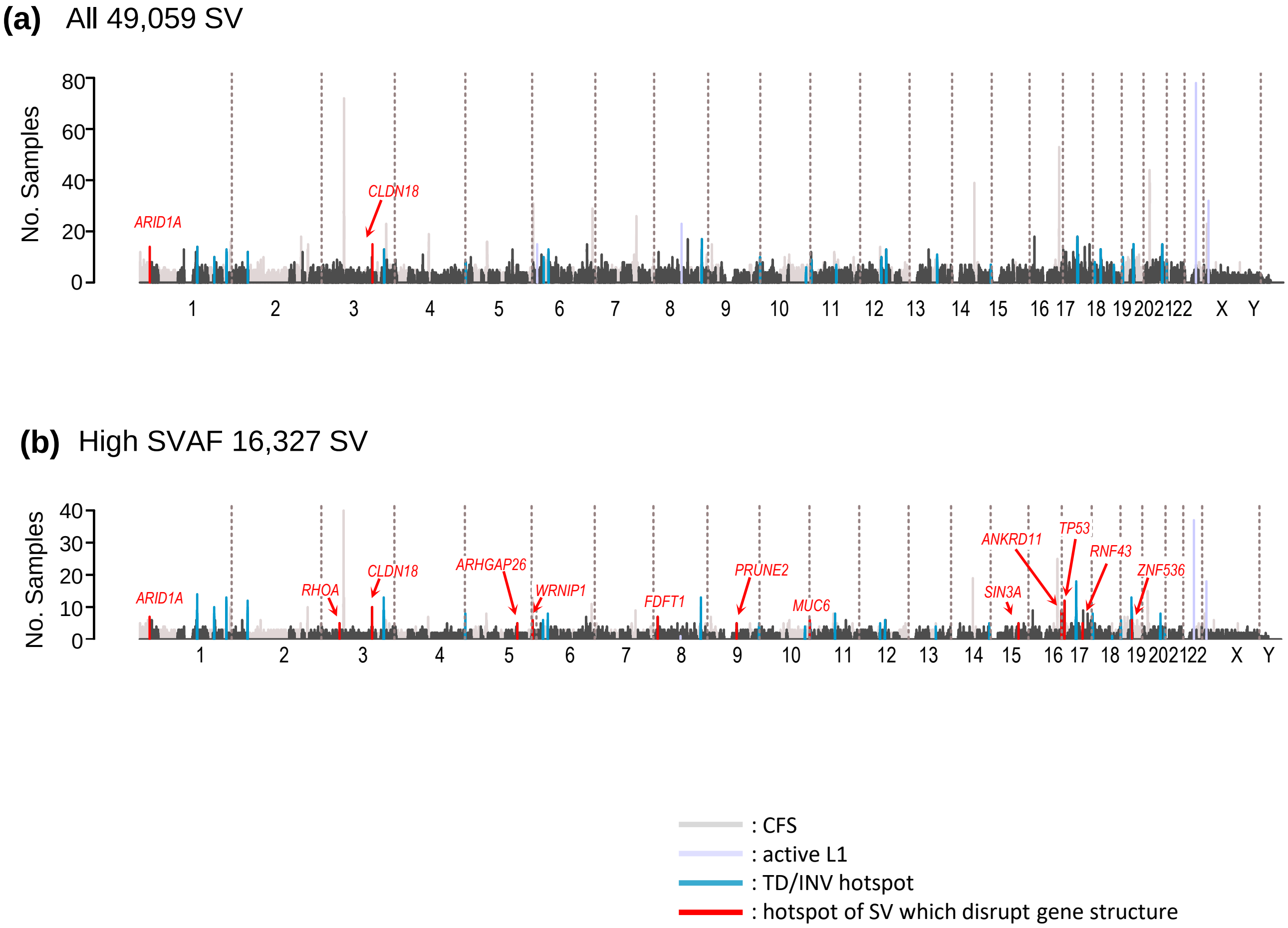

Supplementary Figure 6 Comparison of genomic hotspot distributions between all SVs and high-SVAF SVs.

The distribution of genomic hotspots in all (49,509) (**a**) and those with high SVAF (16,327) (**b**) are shown. We set the threshold of SV hotspot by more than a 10-fold increase of numbers per the 0.1Mb-sized bin compared to the expected (14.3 for all SVs, 4.7 for high SVAF-SVs) and excluding CFS and active LINE1 loci. Among seventy-nine hotspots, 16 had high SVAF SVs. Genes are indicated when the breakpoints of the SVs are concentrated in the coding region. Source data are provided as a Source Data file.

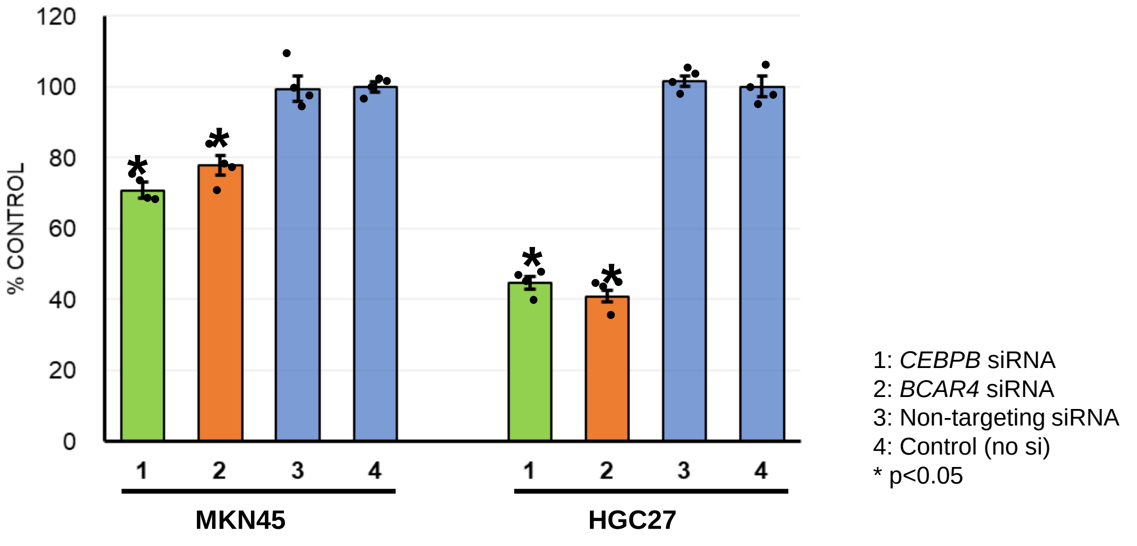

Supplementary Figure 7 Gene knockdown in GC cells.
siRNA knockdown of the CEBPB and BCAR4 significantly reduced cell viability in two GC cells, MKN45 and HGC27. The growth rate percentages were relative to control cells. N=2 biologically independent samples were analysed in four technical replicates. Data points were shown as dots. data are shown as the mean ± SD. Student t-test was used to detect the mean difference. Source data are provided as a Source Data file.

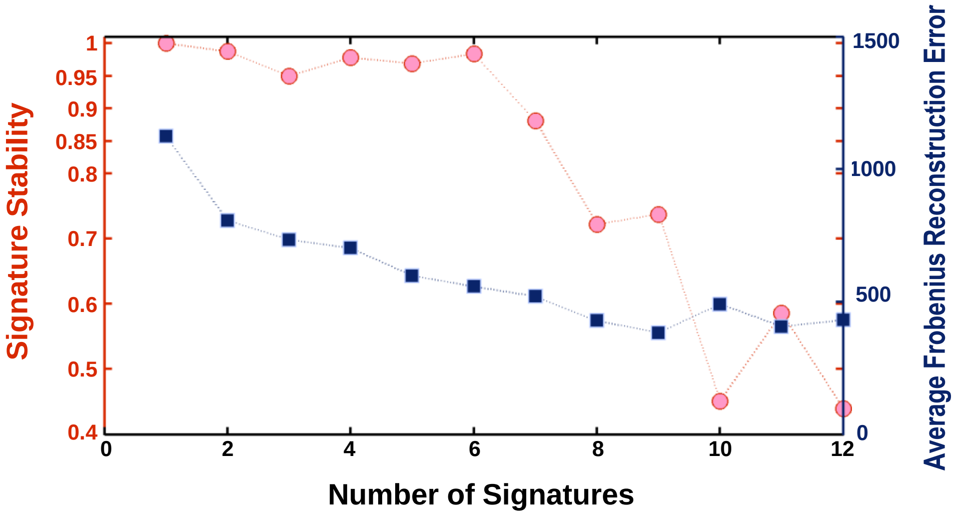

Supplementary Figure 8 Estimation of the number of rearrangement signatures present in 170 GC samples. Non-negative matrix factorization in MATLAB (version 6.1.0.604, The MathWorks, Inc., USA) was used to estimate signature stability and average Frobenius reconstruction error for the analyzed dataset. Signature stability remains high for six signatures extracted, then falls acutely. The Frobenius reconstruction error at that point remains the appropriate value (>500).

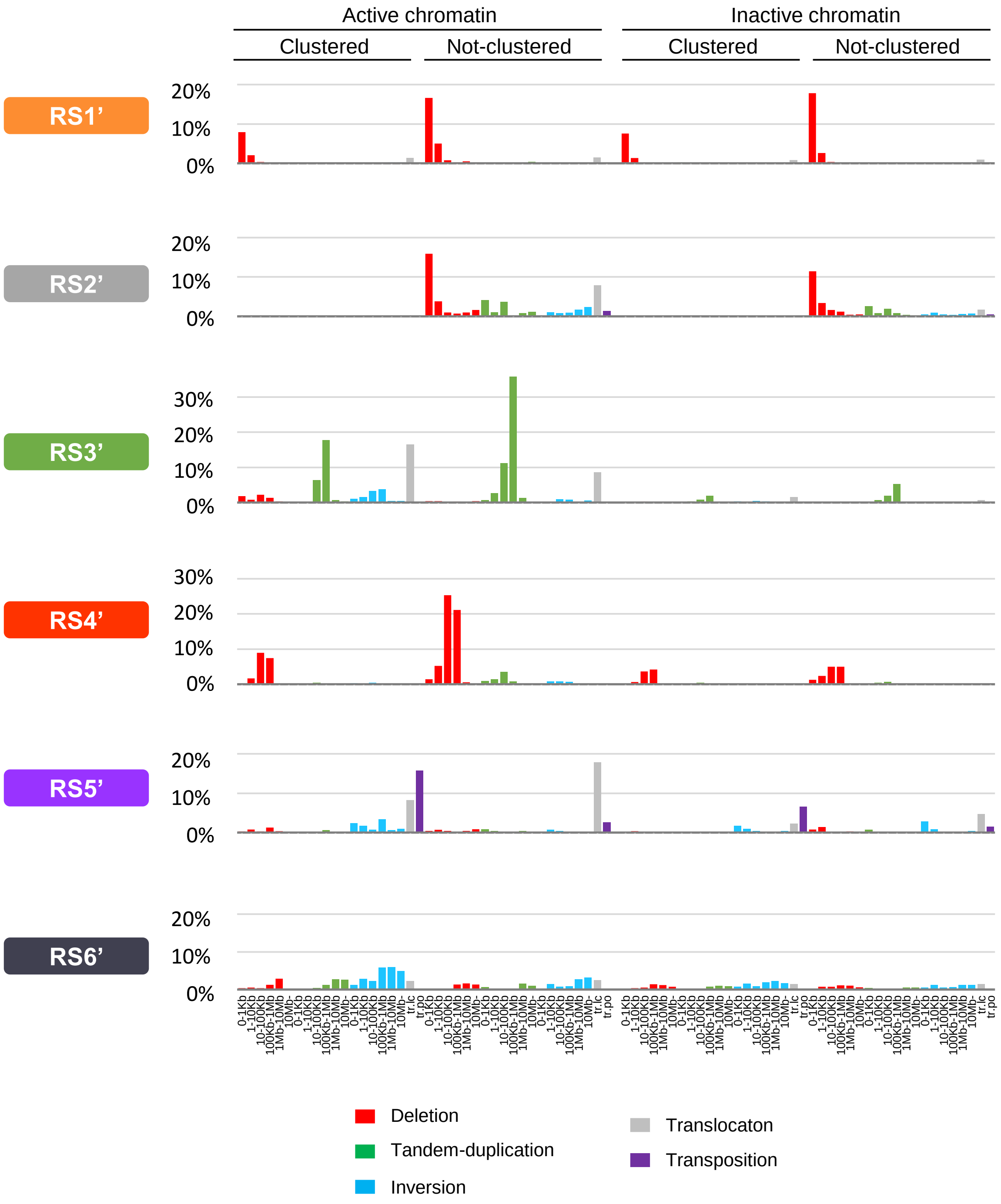

Supplementary Figure 9 The composition of SVs in each RS per 100 Mb
The SV compositions of each RS shown in Figure 2(a) are represented by the frequency per 100 Mb in each chromatin state. Source data are provided as a Source Data file.

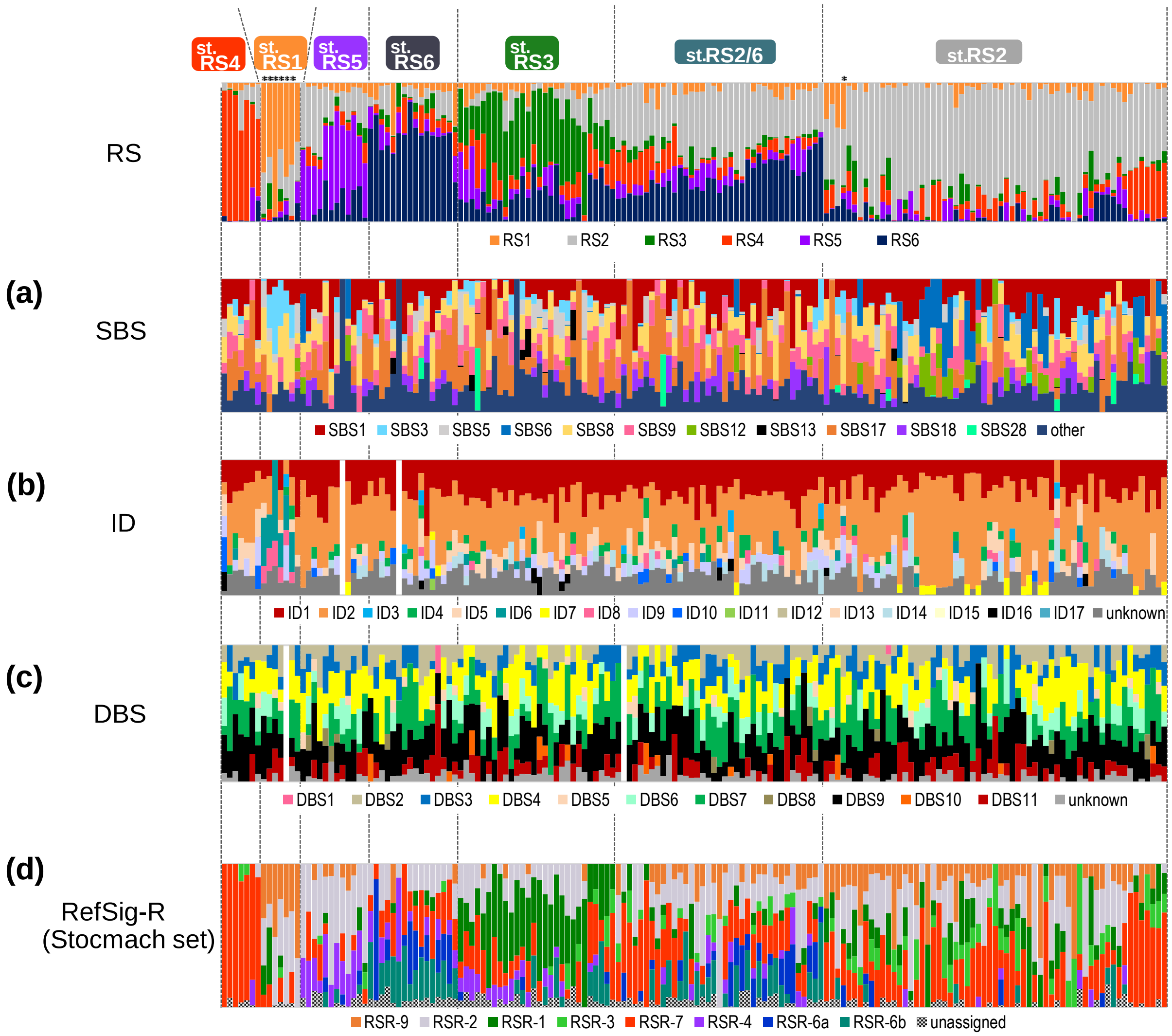

(Figure continued on next page)

(e)

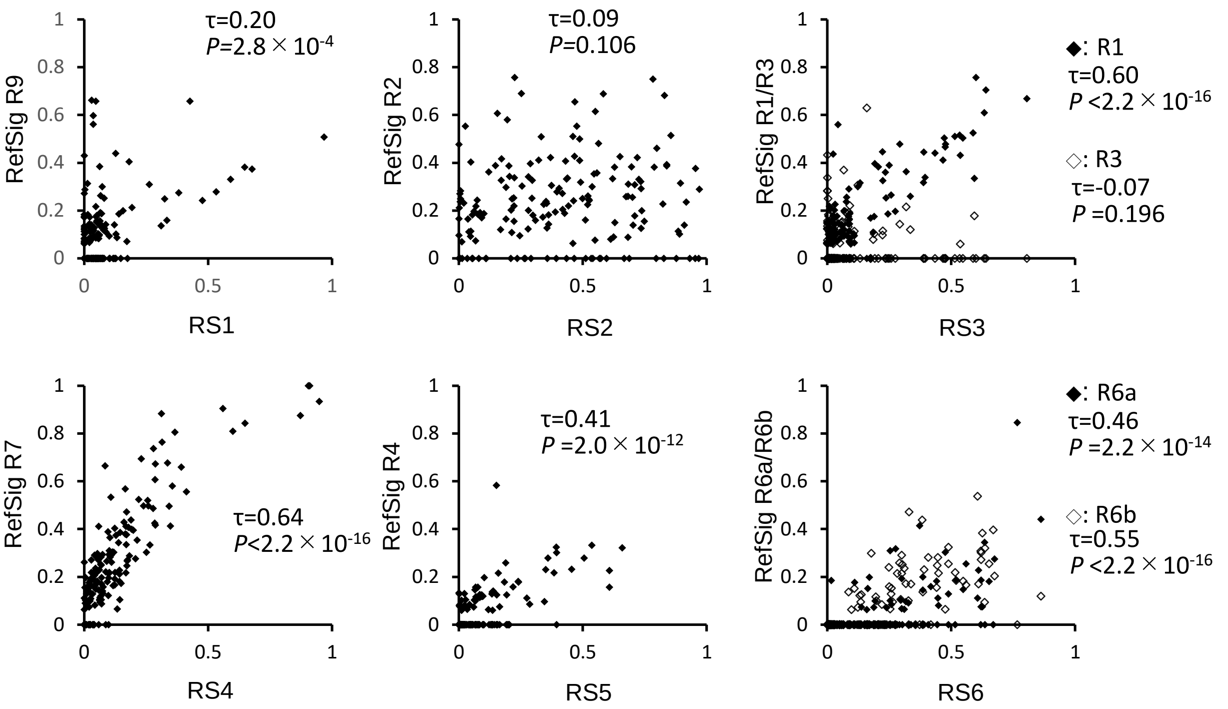

Supplementary Figure 10 Contributions of the COSMIC mutational signatures and the Signal reference RSs in the GC cohort

The asterisk symbols on the RSs contribution plot indicated cases with BRCA phenotypes defined as its SBS3 and ID6 contribution occupied 30% or more.

a-c. Contributions of the three COSMIC mutational signatures; single base substitutions (SBS), small insertions and deletions (ID), and doublet base substitutions (DBS). **d**. Contributions of reference rearrangement signatures[25] (RefSig R, abbreviated as RSR) in stomach cancer, RSR1, RSR2, RSR3, RSR4, RSR6a, RSR6b, RSR7, and RSR9. **e**. The correlations between RS and RSR. Plots showed the RS contribution (vertical axis) and their best-corresponding proposed RSR (horizontal axis). The tau coefficient of correlation and the two-sided P-value is calculated using Kendall's rank correlation test. **a-e,** Source data are provided as a Source Data file.

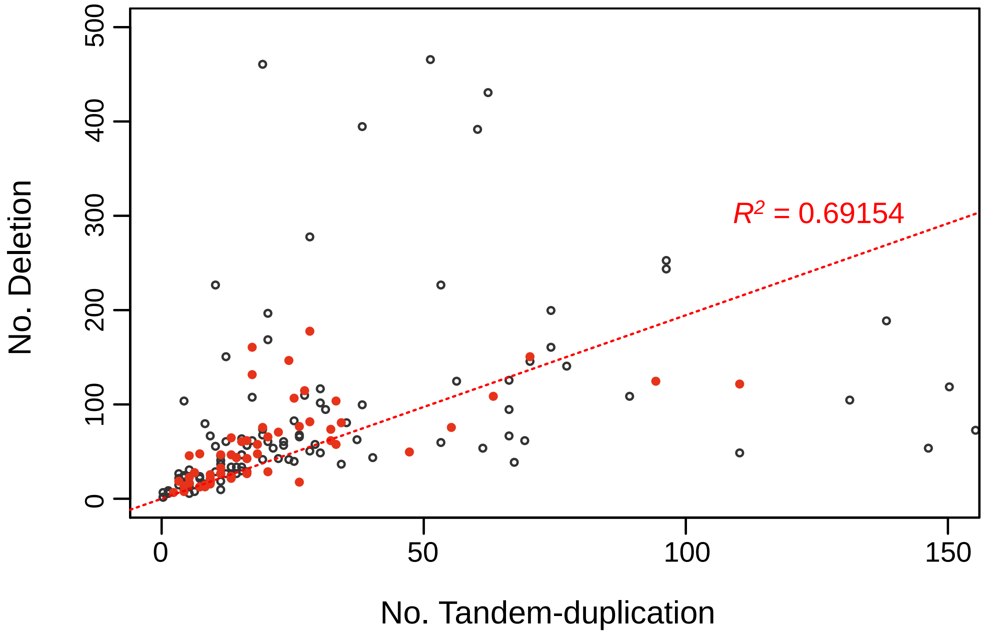

Supplementary Figure 11 Co-occurrence of deletions and tandem duplications
The numbers of small deletions (X-axis) and small tandem duplications (Y-axis) are plotted for each case. Red dots indicate subtype RS2 cases. Source data are provided as a Source Data file.

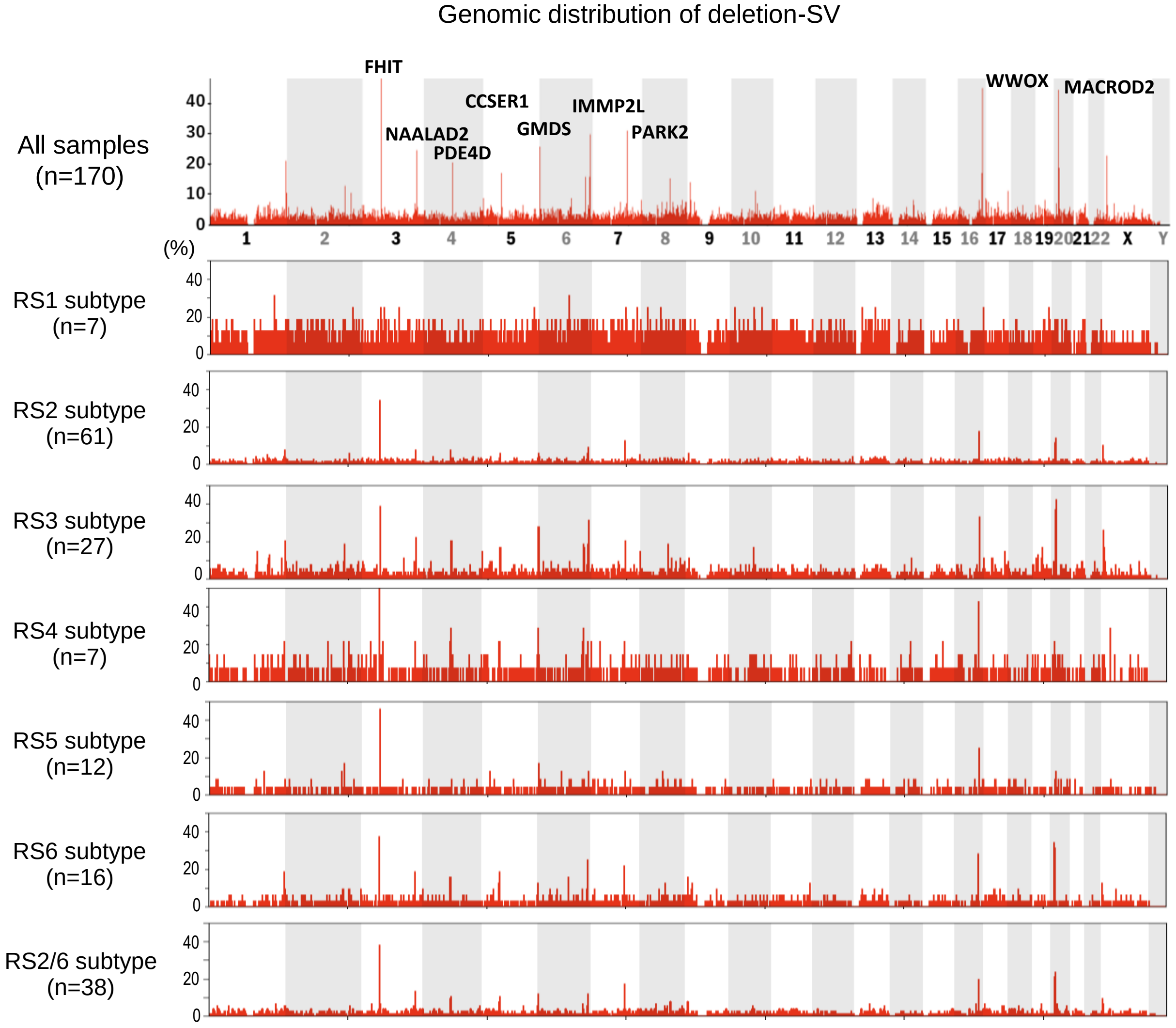

Supplementary Figure 12 Broadly distributed deletions in subtype RS1 cases

The distribution of deletion hotspots was compared for the RS subtype. The seven RS subtypes are abbreviated as st.RS1–st.RS6 and st.RS2/6. Deletions in subtype RS1 spread unbiasedly throughout the genome, whereas those in other subtypes are concentrated at common fragile sites (CFSs). A sliding window of 1 Mb (100 kb overlap) depicted the distribution plots. The distribution of all deletions in the 170 cases and CFSs is shown at the top. Source data are provided as a Source Data file.

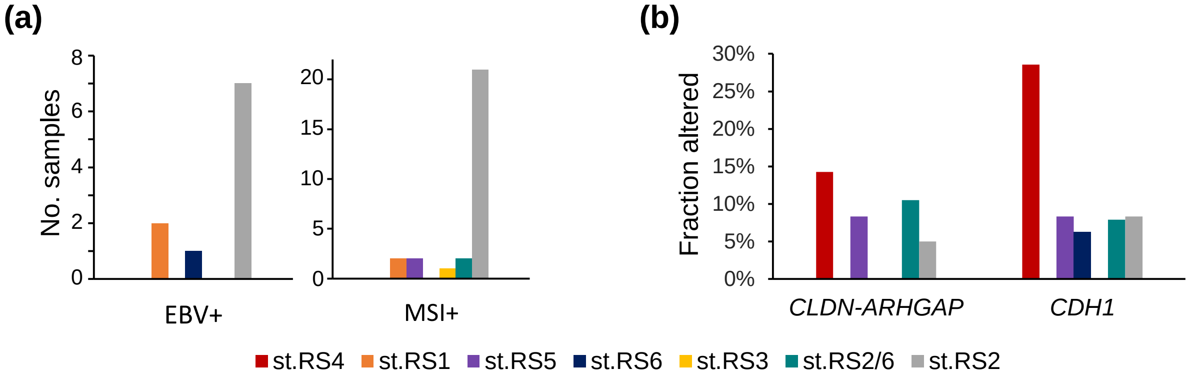

Supplementary Figure 13 Comparison between TCGA GC class and RS subtypes
a. The number of EBV-positive and MSI-positive RS subtypes. **b.** Frequency of diffuse-type GC characteristic drivers, *CLDN–ARHGAP* fusions, and *CDH1* mutations in the RS subtypes. **a-b,** Source data are provided as a Source Data file.

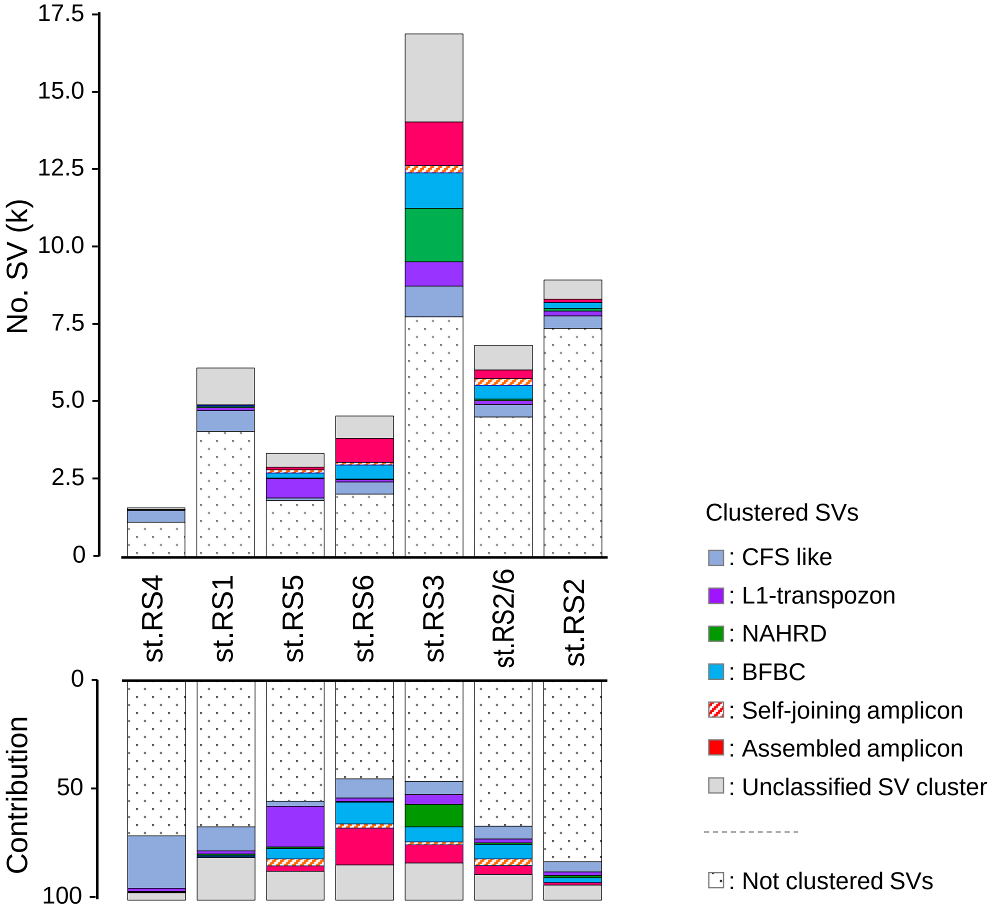

Supplementary Figure 14 The ratio of clustered SVs in RS subtypes.
The numbers and contributions of the clustered SV in each RS subtype are shown. Abbreviations: st., subtype; CFS, common fragile sites; NAHRD, non-allelic homologous recombination-mediated duplications; BFBC, breakage-fusion-bridge cycles. Source data are provided as a Source Data file.

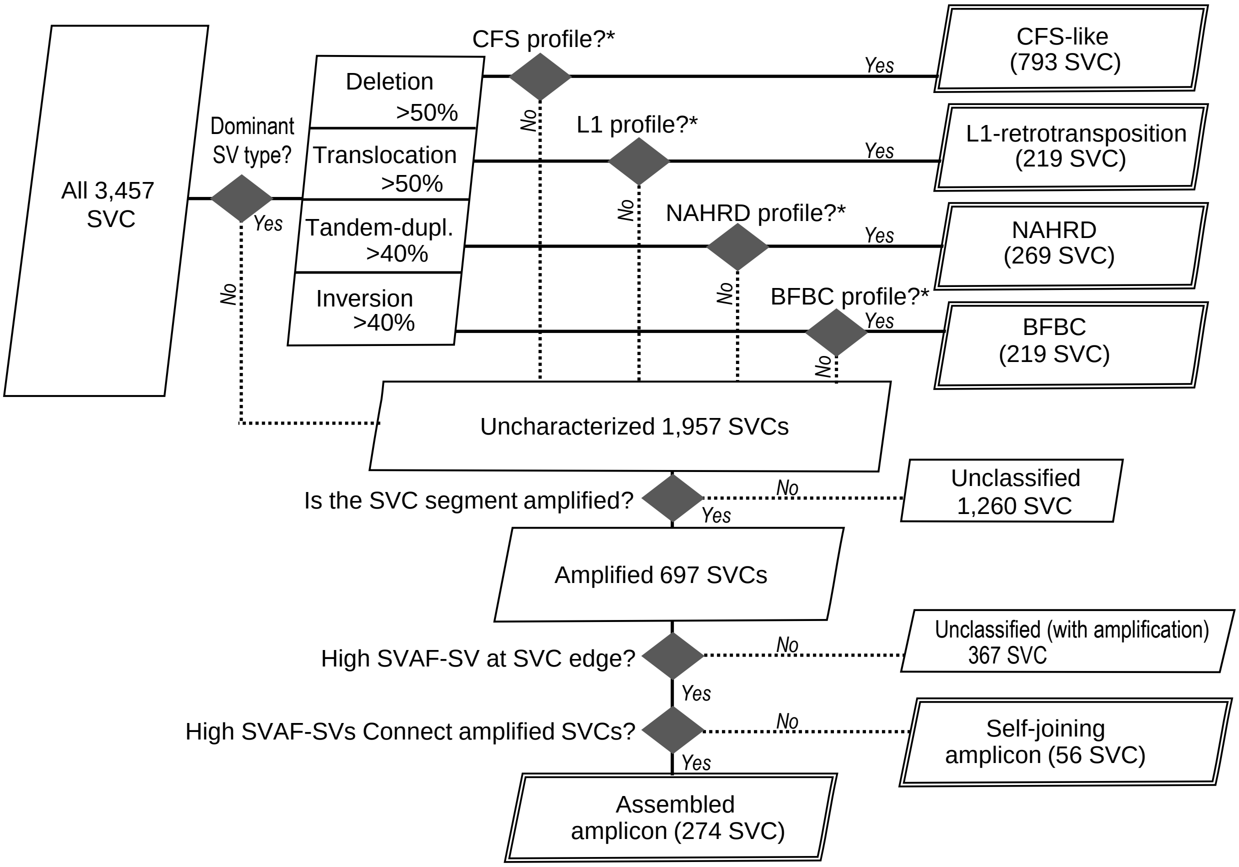

Supplementary Figure 15 Schematic illustration of the 3,457 SVC classification flowchart. The detailed conditions for SV cluster (SVC) classification are abbreviated for chart conciseness. Abbreviations: CFS-like, Common fragile sites; L1: L1-retrotransposon; NAHRD, non-allelic homologous recombination-mediated duplications; BFBC, breakage-fusion-bridge cycles. See the “SVC profile” section in “Methods” for the accurate definition required for each SVC type

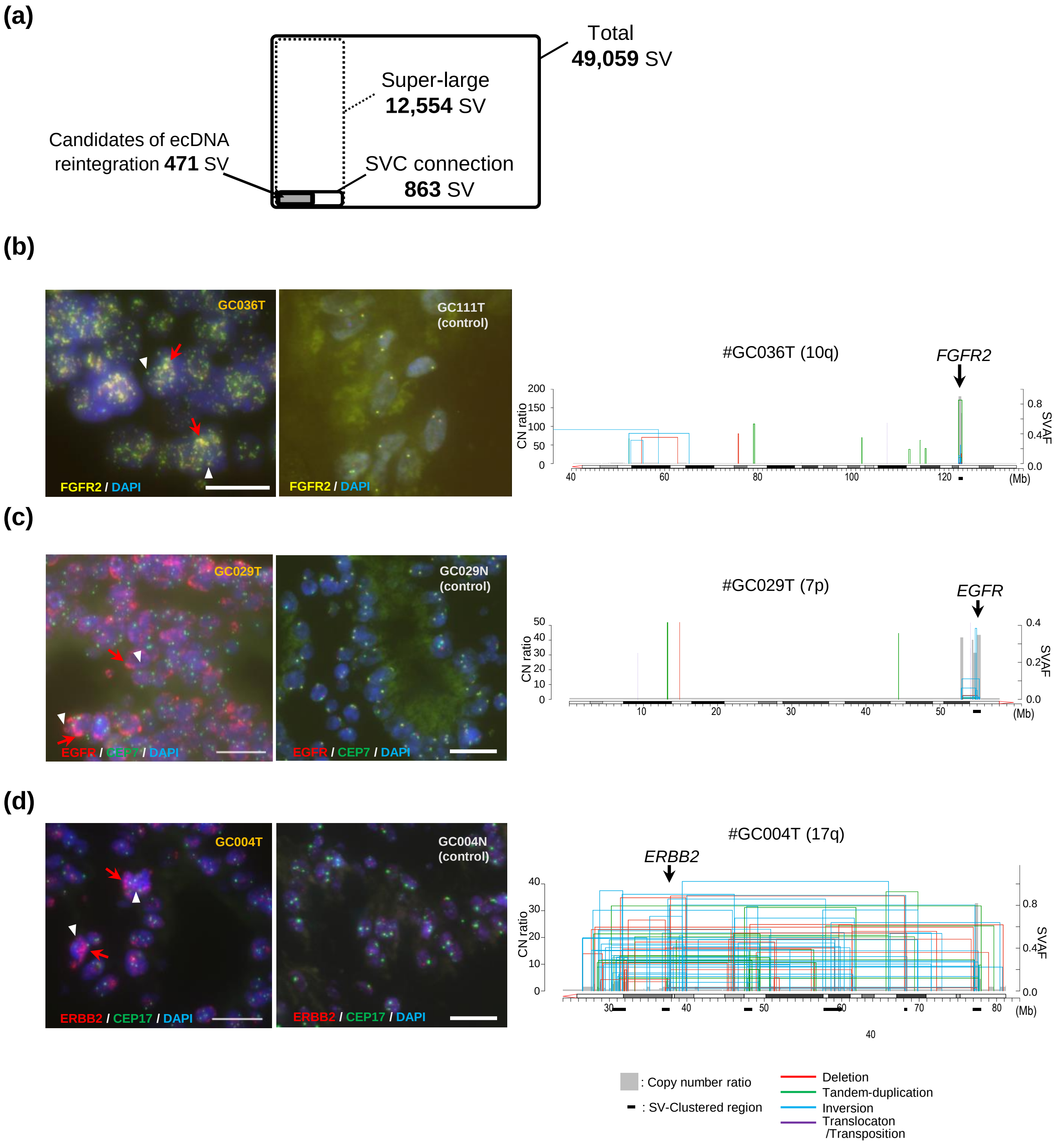

Supplementary Figure 16 Validation of ecDNA and HSR by FISH imaging.
(**a**) Numbers of super-large and SVC connecting SVs and candidates of ecDNA reintegration in this study. (**b–d**) FISH analyses of target gene loci validated ecDNA and HSR. **b**) FGFR2 locus (yellow) in GC036T. **c**) EGFR (red) locus in GC029T. **d**) ERBB2 (red) locus in GC004T. The scattered spots suggest ecDNA (red arrow), and the clustered bright stainings suggest HSR (white arrow). Centromeres of chromosome 7 (EGFR case) and chromosome 17 (ERBB2 case) are shown in green. DAPIs are shown in blue. Control images are shown on the right. Scale bars, 20 μm. **a** and right panels of **b-d**, Source data are provided as a Source Data file.

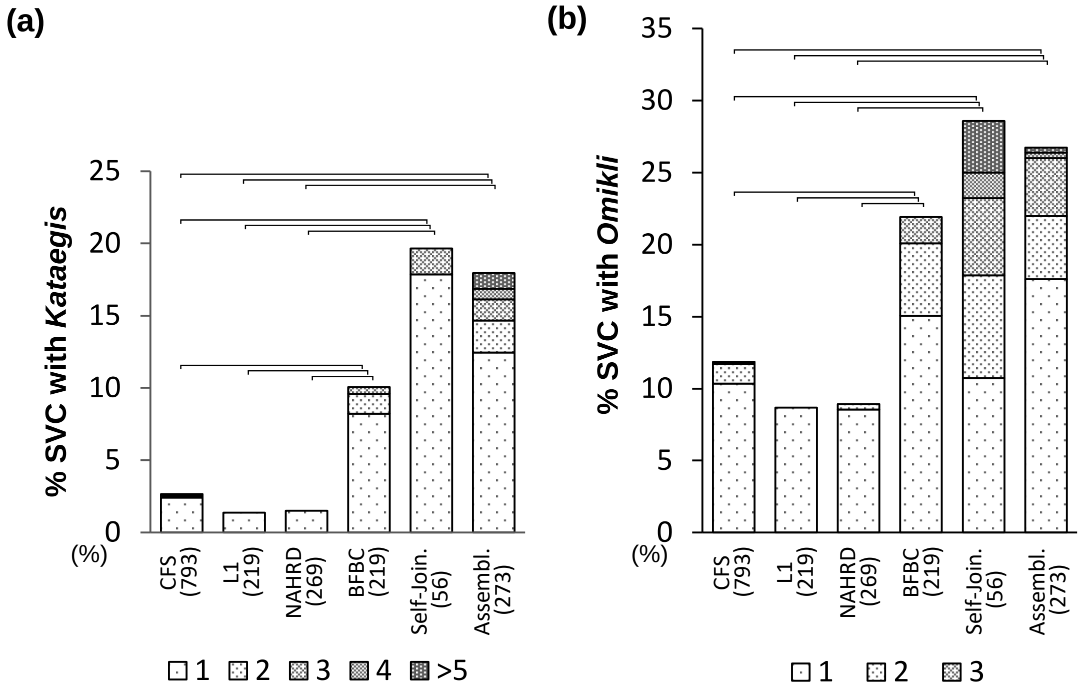

Supplementary Figure 17 The concurrence of SV Cluster and *omikli* and *kataegis*.
For each of the six types of SV cluster (SVC), the bar graph shows the percentage of SVCs in which one or more hypermutation events were observed in that segment. The dotted patterns in the bar indicate the detected numbers of the event from one SVC segment. Lines over bars indicate statistically significant group comparisons using paired two-sided fisher exact test. Abbreviations: CFS, Common fragile sites-like; L1: L1-retrotransposon; NAHRD, non-allelic homologous recombination-mediated duplications, BFBC, breakage-fusion-bridge cycles; Self-Join, self-joining amplicon; and assemble.: assembled amplicons.
a. *Kataegis* (focal hypermutation) in SVCs segments. *P*=1.23e-05, 1.70e-06, and 7.13e-16, respectively, for CFS compared with BFBC, SelfJ, and Assemble. *P*=1.03e-04, 2.34e-06, and 1.42e-10 for L1 compared with BFBC, SelfJ, and Assemble. *P*=2.51e-05, 1.28e-06, and 9.87e-12 for NAHRD compared with BFBC, SelfJ, and Assemble. **b.** *Omikli* (diffuse hypermutation) in SVCs segments. *P*=2.68e-04, 1.34e-03, and 2.44e-08, respectively, for CFS compared with BFBC, SelfJ, and Assemble. *P*=1.68e-04, 2.39e-04, and 2.04e-07 for L1 compared with BFBC, SelfJ, and Assemble. *P*=6.30e-05, 2.06e-04, and 4.79e-08 for NAHRD compared with BFBC, SelfJ, and Assemble.
a-b, Source data are provided as a Source Data file.

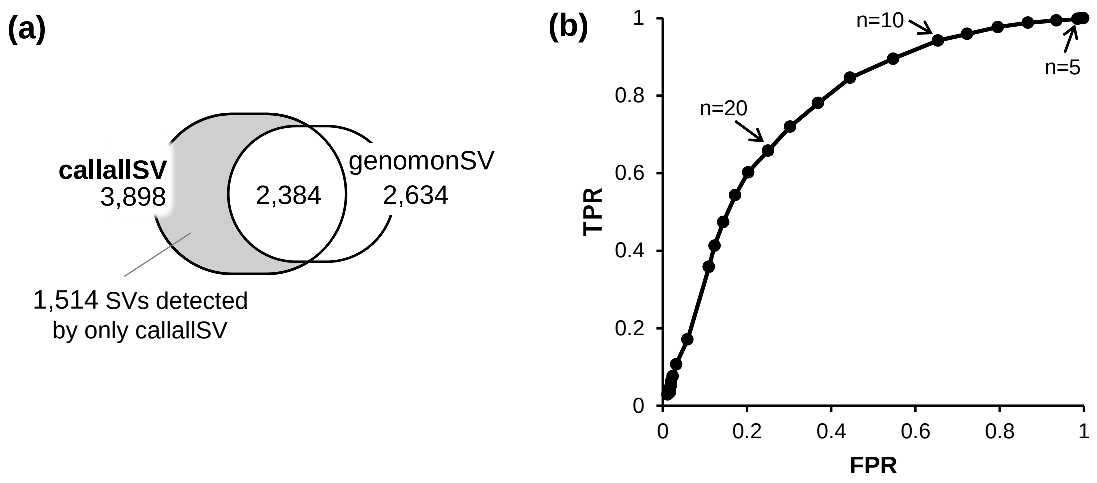

Supplementary Figure 18 The validation of our in-house SV detection pipeline (callallSV).
a. Venn diagram of the validation set comprising 16 cases of GC WGS. Two SV callers, callallSV and GenomonSV (version 2.5.0, https://github.com/Genomon-Project/GenomonSV), detected 3,898 and 2,634 somatic SVs from the dataset, respectively. Of these, 2,384 were commonly detected by both tools, while 1,514 SVs were detected only by callallSV. We randomly selected 56 SVs from these 1,514 SVs uniquely detected by callallSV and validated them using RT-PCR and Sanger DNA sequencing, and 89.3% (50/56) of the SVs were verified. See also **Supplementary Data 12** in detail. **b**. For each number of reads supporting an SV, we calculated the true positive rate (TPR or sensitivity) at which our callallSV detected benchmark SVs and the false positive rate (FPR) at which our callallSV detected non-benchmark SVs. Plotting the ROC curve by these values resulted in an area under the curve of 0.766.

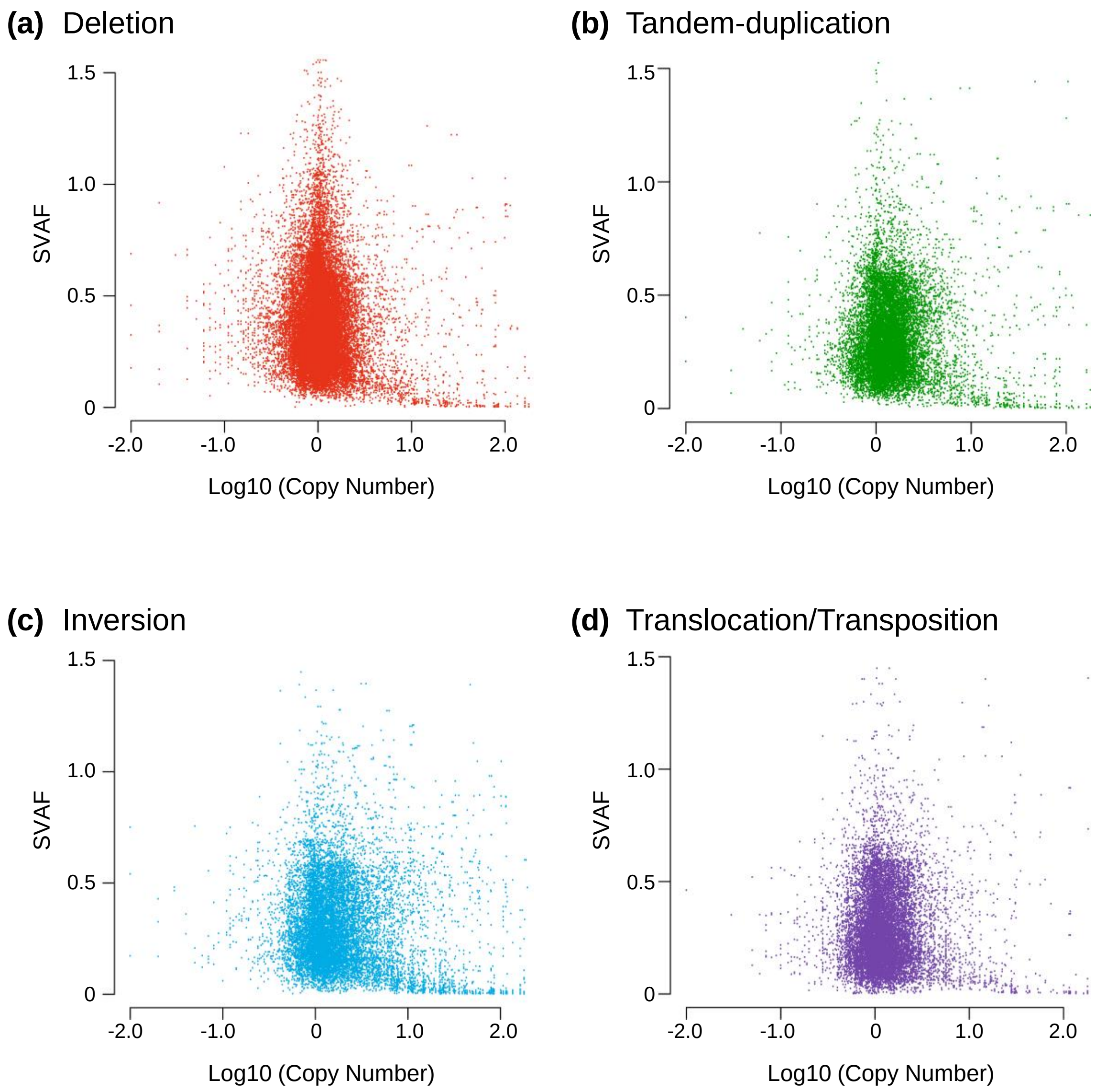

Supplementary Figure 19 The relation between SVAF and the copy number.
Total 48,528 SV (deletion: 21,942; tandem duplication: 11,162; inversion: 8,410; and translocation and transposition: 7,014) was subjected to SVAF calculation, and its SVAF was plotted on the Y-axis, and the log of a segmental copy number at its SV breakpoint on the x-axis. A larger value was used when the copy numbers assigned to two breakpoints of one SV were not identical.

References in Supplementary Data

1. Totoki Y. et al. Multi-ancestry genomic and transcriptomic analysis of gastric cancer. *Nat. Genet.* **55**, 581-594 (2023).
2. Wang, K. et al. Whole-genome sequencing and comprehensive molecular profiling identify new driver mutations in gastric cancer. *Nat. Genet.* **46**, 573–582 (2014).
3. Cancer Genome Atlas Research Network. Comprehensive molecular characterization of gastric adenocarcinoma. *Nature* **513**, 202-9 (2014).
4. Leuzzi G, et al. WRNIP1: A new guardian of genome integrity at stalled replication forks. *Mol Cell Oncol.* **3**, e1215777 (2016).
5. Ha NT. et al. Roles of Farnesyl-Diphosphate Farnesyltransferase 1 in Tumour and Tumour Microenvironments. *Cells* **9**, 2352 (2020).
6. Li T. et al. PRUNE2 inhibits progression of colorectal cancer in vitro and in vivo. *Exp Ther Med.* **23**, 169 (2022).
7. Liu BH. et al. Tumor Suppressive Role of MUC6 in Wilms Tumor via Autophagy-Dependent β-Catenin Degradation. *Front Oncol.* **12**, 756117 (2022).
8. Lewis MJ. et al. SIN3A and SIN3B differentially regulate breast cancer metastasis. *Oncotarget.* **7**, 78713-78725 (2016).
9. Lim SP. et al. Specific-site methylation of tumour suppressor ANKRD11 in breast cancer. *Eur J Cancer* **48**, 3300-3309 (2012).
10. Xu, Xintong. Analysis of the Target Genes of Transcription Factor ZNF536 in Lung Adenocarcinoma. *ICBBT'19: Proceedings of the 2019 11th International Conference on Bioinformatics and Biomedical Technology.* 81-85 (2019)
11. Li L. et al. Genome-scale CRISPRa screening identifies MTX1 as a contributor for sorafenib resistance in hepatocellular carcinoma by augmenting autophagy. *Int J Biol Sci.* **17**, 3133-3144 (2021).
12. Wang W. et al. LINC00184 plays an oncogenic role in non-small cell lung cancer via regulation of the miR-524-5p/HMGB2 axis. *J Cell Mol Med.* **25**, 9927-9938 (2021).
13. Wu R.C. et al. Identification of the PTEN-ARID4B-PI3K pathway reveals the dependency on ARID4B by PTEN-deficient prostate cancer. *Nat. Commun.* **10**, 4332 (2019).
14. Xing R. et al. Whole-genome sequencing reveals novel tandem-duplication hotspots and a prognostic mutational signature in gastric cancer. *Nat. Commun.* **10**, 2037 (2019).
15. Zhu X. et al. Targeting BRD9 for Cancer Treatment: A New Strategy. *Onco Targets Ther.* **13**, 13191-13200 (2020).
16. Liu X. et al. MDC1 promotes ovarian cancer metastasis by inducing epithelial-mesenchymal transition. *Tumour Biol.* **36**, 4261-4269 (2015)
17. Kim M. et al. VEGFA links self-renewal and metastasis by inducing Sox2 to repress miR-452, driving Slug. *Oncogene* **36**, 5199-5211(2017).
18. Renehan A.G. et al. Obesity and cancer risk: the role of the insulin-IGF axis. *Trends Endocrinol Metab.* **17**, 328-336 (2006).
19. Zhang X. et al. Somatic Superenhancer Duplications and Hotspot Mutations Lead to Oncogenic Activation of the KLF5 Transcription Factor. *Cancer Discov.* **8**, 108-125 (2018).
20. Xing Z. et al. lncRNA directs cooperative epigenetic regulation downstream of chemokine signals. *Cell* **159**, 1110-1125 (2014).
21. Belaguli N.S. et al. GATA6 promotes colon cancer cell invasion by regulating urokinase plasminogen activator gene expression. *Neoplasia* **12**, 856-865 (2010).
22. Gordon-Weeks A. et al. Tumour-Derived Laminin 5 (LAMA5) Promotes Colorectal Liver Metastasis Growth, Branching Angiogenesis and Notch Pathway Inhibition. *Cancers (Basel)* **11**, 630 (2019).
23. Nik-Zainal S. et al. Landscape of somatic mutations in 560 breast cancer whole-genome sequences. *Nature* **534**, 47-54 (2016).
24. Degasperi, A. et al. A practical framework and online tool for mutational signature analyses show intertissue variation and driver dependencies. *Nat. Cancer* **1**, 249–263 (2020).
25. Li Y. et al. Patterns of somatic structural variation in human cancer genomes. *Nature* **578**, 112-121 (2020).
