# Supplementary material for: Oncogenic structural aberration landscape in gastric cancer genomes
Source: Nat Commun. 2023 Jun 22;14:3688. doi: 10.1038/s41467-023-39263-1 (PMC10287692; doi:10.1038/s41467-023-39263-1)
Supplement: Supplementary file 3 — Description to Additional Supplementary Files [file 41467_2023_39263_MOESM3_ESM.pdf]

Supplementary Data Files

Supplementary Data 1 Genomic variants and clinical data of 170 gastric cancer (GC) samples in the study

Supplementary Data 2 The frequency of GC mutation signatures in the chromatin active or inactive area

Supplementary Data 3 Significantly dominant single-base substitution signatures in active or inactive areas in non-hyper-mutated cases

Supplementary Data 4 List of 49,059 somatic structural variants detected in 170 GC samples

Supplementary Data 5 Twelve hotspots of SVs that disrupt gene structure

Supplementary Data 6 Twenty-seven hotspots of SVs that amplified oncogene

Supplementary Data 7 Comparison with previous studies on structural rearrangement signatures

Supplementary Data 8 Correlation between the counts of *omikli* and *kataegis* events and rearrangement signatures

Supplementary Data 9 Criteria for structural variant cluster (SVC) profiles

Supplementary Data 10 The proportion of specific SVs in each type of SVC

Supplementary Data 11 The SV candidates of ecDNA reintegration into chromosomal DNA

Supplementary Data 12 PCR primer sequences in validation analysis of 123 SVs

Supplementary Data 13 siRNA and qPCR primers
