# Supplementary material for: Oncogenic structural aberration landscape in gastric cancer genomes
Source: Nat Commun. 2023 Jun 22;14:3688. doi: 10.1038/s41467-023-39263-1 (PMC10287692; doi:10.1038/s41467-023-39263-1)
Supplement: Supplementary file 5 — Reporting Summary [file 41467_2023_39263_MOESM5_ESM.pdf]

nature portfolio

Corresponding author(s): Tatsuhiro Shibata

Last updated by author(s): May 14, 2023

Reporting Summary

Nature Portfolio wishes to improve the reproducibility of the work that we publish. This form provides structure for consistency and transparency in reporting. For further information on Nature Portfolio policies, see our Editorial Policies and the Editorial Policy Checklist.

Statistics

For all statistical analyses, confirm that the following items are present in the figure legend, table legend, main text, or Methods section.

n/a	Confirmed	
☐	☒	The exact sample size (*n*) for each experimental group/condition, given as a discrete number and unit of measurement
☒	☐	A statement on whether measurements were taken from distinct samples or whether the same sample was measured repeatedly
☐	☒	The statistical test(s) used AND whether they are one- or two-sided<br>*Only common tests should be described solely by name; describe more complex techniques in the Methods section.*
☒	☐	A description of all covariates tested
☒	☐	A description of any assumptions or corrections, such as tests of normality and adjustment for multiple comparisons
☐	☒	A full description of the statistical parameters including central tendency (e.g. means) or other basic estimates (e.g. regression coefficient) AND variation (e.g. standard deviation) or associated estimates of uncertainty (e.g. confidence intervals)
☐	☒	For null hypothesis testing, the test statistic (e.g. *F*, *t*, *r*) with confidence intervals, effect sizes, degrees of freedom and *P* value noted<br>*Give P values as exact values whenever suitable.*
☒	☐	For Bayesian analysis, information on the choice of priors and Markov chain Monte Carlo settings
☒	☐	For hierarchical and complex designs, identification of the appropriate level for tests and full reporting of outcomes
☐	☒	Estimates of effect sizes (e.g. Cohen's *d*, Pearson's *r*), indicating how they were calculated

Our web collection on statistics for biologists contains articles on many of the points above.

Software and code

Policy information about availability of computer code

Data collection	No commercial software was used for data collection. All of the software used is reported in the paper.
Data analysis	MATLAB (version 6.1.0.604, The MathWorks, Inc., USA) was used for non-negative matrix factorization.<br>Open sources used to analyze the data in this study are the following.<br>BWA-MEM (version 0.7.8): Mapping short-read sequences against a large reference genome, such as the human genome.<br>NovoAlign (version 3.2.8): Mapping of short reads onto a reference genome.<br>SAMtools (version 1.9): Remove PCR duplications and generate pileup files from mapped BAM files.<br>deconstructSigs (version 1.8.0): Determine the weights of each mutational signature contributing to an individual tumor sample.<br>FACETS (version 0.6.0): Allele-specific copy number and clonal heterogeneity analysis tool for high-throughput DNA sequencing.<br>SigProfilerClusters (Version 1.0.0): Analyzing the inter-mutational distances between SNV-SNV and INDEL-INDEL mutations<br>DNACopy (version 1.56.0): Segmentation for the analysis of DNA copy number data.<br>Fusionfusion (version 0.3.0): Detecting gene fusion using the putative chimeric transcript generated by several well-known transcriptome alignment tools (STAR, MapSplice2, and TopHat2).<br>STAR (version 2.5.2a): Ultrafast universal RNA-seq aligner.

For manuscripts utilizing custom algorithms or software that are central to the research but not yet described in published literature, software must be made available to editors and reviewers. We strongly encourage code deposition in a community repository (e.g. GitHub). See the Nature Portfolio guidelines for submitting code & software for further information.

Data

Policy information about availability of data

All manuscripts must include a data availability statement. This statement should provide the following information, where applicable:

- Accession codes, unique identifiers, or web links for publicly available datasets
- A description of any restrictions on data availability
- For clinical datasets or third party data, please ensure that the statement adheres to our policy

All data needed to evaluate the conclusions in this study are presented in this paper, the Supplementary Materials, or are available at the following repository. The WGS data of 81 Japanese cases with their prefix 'GC' in this paper have been deposited in European Genome-phenome Archive (https://ega-archive.org/) with the accession numbers EGAD00001008610 [https://ega-archive.org/datasets/EGAD00001008610] and EGAS00001006051 [https://ega-archive.org/studies/EGAS00001006051]. Requests for academic purposes only will be processed by ICGC Data Access Compliance Office (https://docs.icgc-argo.org/docs/data-access/daco/applying) within ten business days. After access has been granted, the data is available for two years. The RNA sequencing data of the 62 Japanese cases generated in previous study4 are available in the Japanese Genotype-phenotype Archive with the accession codes JGAS000228 [https://humandbs.biosciencedbc.jp/en/hum0227-v1] and JGAS000229 [https://humandbs.biosciencedbc.jp/en/hum0226-v1]. For use, approval is required at the review by the NBDC Human Data Review Board. Data users shall apply for data use in accordance with the data use application procedures (https://humandbs.biosciencedbc.jp/en/data-use). These data are under controlled access because they are personally identifiable data defined by Japan's Personal Information Protection Law.
The WGS data of 89 Chinese cases generated in previous study19 with their prefix 'pfg' have been deposited in EGAD00001000782 [https://ega-archive.org/datasets/EGAD00001000782] and EGAS00001000597 [https://ega-archive.org/studies/EGAS00001000597]. The datasets are available under controlled access and access can be obtained by contacting the University of Hong Kong Gastric Cancer Genomics Study Data Access Committee. The remaining data are available within the Article, Supplementary Information or Source Data file.

Research involving human participants, their data, or biological material

Policy information about studies with human participants or human data. See also policy information about sex, gender (identity/presentation), and sexual orientation and race, ethnicity and racism.

Reporting on sex and gender	The participants for this study consists of 109 males and 61 females. For greater security and privacy, we decided not to provide gender information in each case.
Reporting on race, ethnicity, or other socially relevant groupings	Individuals were categorised into two ethnic categories representing distinct genetic ancestries: Eighty-one Japanese GC cases and eighty-nine Chinese cases.
Population characteristics	The age at diagnosis of 170 participants ranged from late 30s to late 80s, and the mean was 69. See Supplementary Table 1 for detail.
Recruitment	Gastric cancer patients in National Cancer Center (Tokyo, Japan) were recruited with consent to provide their surgical sections for academic studies. Queen Mary Hospital, The University of Hong Kong (Pokfulam, Hong Kong) were recruited with consent. There is no potential self-selection or other biases in this work.
Ethics oversight	The study was approved by the National Cancer Center Ethical Commitee (G20-03).

Note that full information on the approval of the study protocol must also be provided in the manuscript.

Field-specific reporting

Please select the one below that is the best fit for your research. If you are not sure, read the appropriate sections before making your selection.

☒ Life sciences ☐ Behavioural & social sciences ☐ Ecological, evolutionary & environmental sciences

For a reference copy of the document with all sections, see nature.com/documents/nr-reporting-summary-flat.pdf

Life sciences study design

All studies must disclose on these points even when the disclosure is negative.

Sample size	No sample size caluculation was performed because the present study is observational, not interventional.
Data exclusions	Detailes of the exclusion criteria are reported in the paper. Samples not passing quality-contorol have been excluded.
Replication	Not applicable for this observational study.
Randomization	Not applicable for this observational study.
Blinding	Not applicable for this observational study.

Reporting for specific materials, systems and methods

We require information from authors about some types of materials, experimental systems and methods used in many studies. Here, indicate whether each material, system or method listed is relevant to your study. If you are not sure if a list item applies to your research, read the appropriate section before selecting a response.

Materials & experimental systems

n/a	Involved in the study
☒	☐ Antibodies
☐	☒ Eukaryotic cell lines
☒	☐ Palaeontology and archaeology
☒	☐ Animals and other organisms
☒	☐ Clinical data
☒	☐ Dual use research of concern
☒	☐ Plants

Methods

n/a	Involved in the study
☒	☐ ChIP-seq
☒	☐ Flow cytometry
☒	☐ MRI-based neuroimaging

Eukaryotic cell lines

Policy information about cell lines and Sex and Gender in Research

Cell line source(s)	MKN45 (RCB1001), Poorly differentiated gastric adenocarcinoma; HGC27 (RCB0500), Undifferentiated carcinoma derived from human metastatic lymph node of gastric cancer.
Authentication	Cell lines were obtained from Riken Cell Bank, and therefore were not authentificated.
Mycoplasma contamination	All cell lines were tested negative for mycoplasma.
Commonly misidentified lines (See ICLAC register)	None of the cell lines is listed in ICLAC database (v12) of misidentified cell lines.
